# Insights into the gut microbiome-metabolite dynamics in breast cancer

**DOI:** 10.1080/29933935.2025.2483446

**Published:** 2025-04-09

**Authors:** Haseeb Manzoor, Masood Ur Rehman Kayani

**Affiliations:** Metagenomics Discovery Lab, School of Interdisciplinary Engineering & Sciences (SINES), National University of Sciences & Technology (NUST), Islamabad, Pakistan

**Keywords:** Breast cancer, gut microbiome, gut microbial metabolites, anti-cancer properties, pro-cancer, breast cancer treatment, precision oncology

## Abstract

In recent years, understanding the intricate connection between gut microbiome and cancer development has gained significant attention. The gut microbiome has a key role in maintaining overall human health and modulating the body’s defense mechanism against various diseases. This review examines the multifaceted association between the gut microbiome and breast cancer, providing a comprehensive overview of studies from the last two decades that investigate both anti-cancer and pro-cancer properties of gut metabolites. Compounds such as nisin, inosine, acetate, propionate, and conjugated linoleic acids have demonstrated potential as therapeutic agents against breast cancer, while others, including butyrate, lactate, certain bile acids, and secondary metabolites, exhibit dual roles, showing both anti-cancer and pro-cancer properties under different conditions, with some implicated in tumor progression. Moreover, emerging research highlights the dual roles of these metabolites in influencing the efficacy of conventional breast cancer therapies. Despite promising evidence, the molecular mechanisms underlying these opposing actions remain unclear and require further investigation. To advance our understanding, future research should prioritize elucidating these mechanisms, establishing dose-response relationships, and conducting animal and clinical studies to validate *in vitro* findings. This review also identifies key gaps and highlights potential directions for future research in this field.

## GRAPHICAL ABSTRACT

**Figure:**
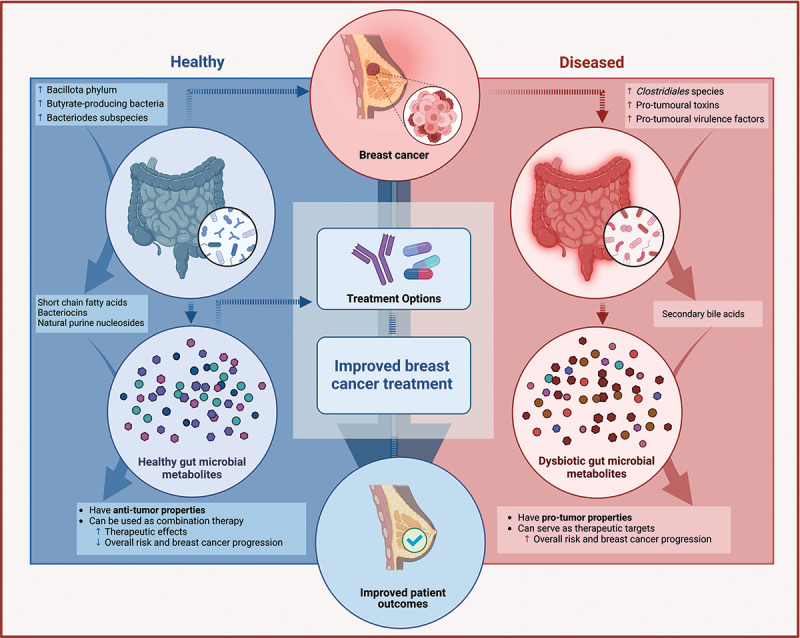

## Introduction

Breast cancer remains one of the leading causes of mortality among women worldwide despite significant advancements in clinical research and treatment strategies.^7^ In December 2020, the International Agency for Research on Cancer (IARC) reported that breast cancer had surpassed lung cancer as the most commonly diagnosed cancer globally. According to the World Health Organization (WHO), 2.3 million new cases of breast cancer and 685,000 deaths were recorded that year.^7^ Alarming projections indicate that by 2040, the global incidence of breast cancer could increase by 40% to reach 3 million new cases annually, while the mortality rate may surpass 1 million deaths, reflecting a 50% surge.^7^ These concerning trends highlight the urgent need for innovative and effective strategies in breast cancer prevention and treatment.

**Figure 1.**
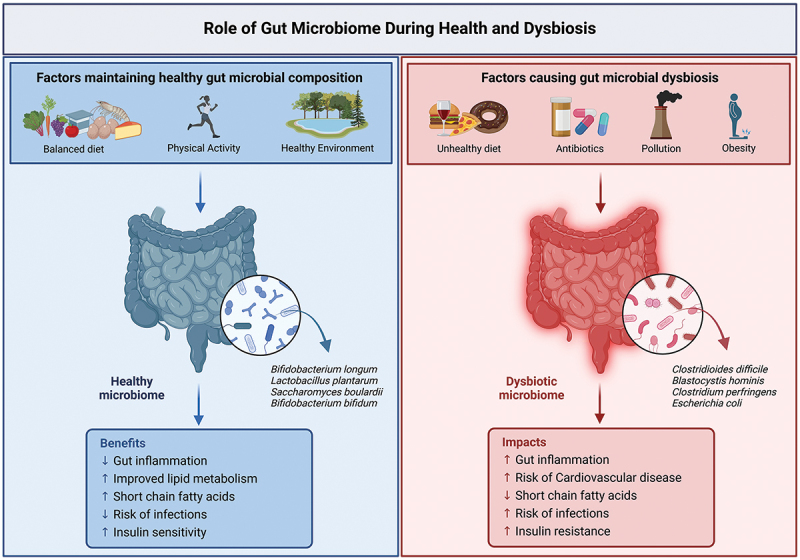
The role of the gut microbiome during health and dysbiosis. A balanced diet, physical activity, and a healthy environment promote a healthy microbiome, enriched with beneficial species like *Bifidobacterium* and *Lactobacillus*, which support gut health, lipid metabolism, and immunity. In contrast, an unhealthy diet, antibiotics, pollution, and obesity contribute to dysbiosis, characterized by harmful species like *Clostridioides difficile* and *Escherichia coli*, leading to inflammation, cardiovascular risks, and insulin resistance.

Recent research has identified the gut microbiome and its metabolic by-products, known as gut metabolites, as potential modulators of breast cancer risk, progression, and therapeutic response. The gut microbiome is comprised of a diverse community of microorganisms, including bacteria, viruses, fungi, and archaea, residing in the gastrointestinal system.^51^ A healthy human gut hosts 300 to 500 microbial species, predominantly from the Actinomycetota, Bacteroidota, Pseudomonadota, and Bacillota phyla.^14^ These microorganisms are crucial for maintaining gut homeostasis, supporting immune function, and influencing overall health.^51^ However, the composition of the gut microbiome is dynamic and is influenced by genetic, dietary, environmental, and lifestyle factors, making it unique to each individual.^111^ When this balance is disrupted, a condition known as gut dysbiosis has been linked to various metabolic, inflammatory, and autoimmune diseases, as well as cancer^56^ (Figure 1).

The gut microbiome plays a crucial role in carcinogenesis, interacting dynamically with the human genome and immune system to influence disease susceptibility. Dysbiosis contributes to cancer development through multiple mechanisms, including the production of mutagenic metabolites, alterations in stem cell dynamics, enhanced cell proliferation, and chronic inflammation.^113^ Notably, specific pathogens have been associated with certain types of cancer, such as *Helicobacter pylori* in stomach cancer,^100^ *Chlamydia trachomatis* in cervical cancer,^134^ *Salmonella typhi* in gall bladder cancer,^60^ and *Fusobacterium nucleatum* and *Bacteroides fragilis* in colorectal cancer^109,143^

Beyond its association with gastrointestinal malignancies, the gut microbiome is increasingly recognized as a key factor in breast cancer development and progression. It can influence the hallmarks of cancer described by Hanahan and Weinberg, which define how a normal cell transforms into a malignant one within the tumor microenvironment.^35^ Additionally, polymorphic microbes themselves are now being considered defining features of cancer.^35^ Furthermore, gut microbiome-derived metabolites exhibit both anti-cancer and pro-cancer properties, influencing immune modulation, estrogen metabolism, inflammation, and tumor suppression.^93^ Emerging studies highlight the potential of these microbial by-products in shaping disease outcomes and modulating breast cancer progression.^93^

This review aims to provide comprehensive insights into the gut microbiome-metabolite interactions in breast cancer, examining their mechanistic roles, therapeutic potential, and implications for future research. A deeper understanding of this intricate relationship may open new avenues for microbiome-targeted breast cancer prevention and treatment strategies.

## Association of gut microbiome and breast cancer

The association between breast cancer and gut microbiome has gained significant attention in recent years, with studies suggesting that microbiome alterations may play a role in disease development and progression.^130^ Distinct microbial signatures have been identified in breast tissue, some of which share similarities with the gut microbiome.^26^ However, it remains unclear whether the microbiome contributes to breast cancer development or is merely an associated phenomenon.^32^ To explore this relationship, key studies from the past decade have been summarized in Table 1, which were selected based on keyword searches such as “gut microbiome,” “gut microbiota,” and “breast cancer.” Studies that were retracted, irrelevant, or lacked direct discussion of the gut microbiome in the context of breast cancer were excluded.

**Table 1.** Studies addressing the association between breast cancer and gut microbiome.

Variations in Gut Microbiome	Sample Type and Cohort	Methodology	Reference
Stage I BC showed higher *Blautia spp*. ratios than stage III, with significant differences in *Bifidobacterium*, *Blautia*, and *Faecalibacterium prausnitzii* across stages.	Fecal samples from 32 BC women (81% invasive ductal, 46.9% stage 0, 62.5% grade II, 80% ER/PgR+, 15% HER2+)	16S rRNA sequencing	Bard et al.^11^
BC patients showed higher *Clostridiaceae*, *Faecalibacterium*, and *Ruminococcaceae* but lower *Dorea* and *Lachnospiraceae* ratios compared to controls.	Fecal and urine samples from 48 postmenopausal BC women and 48 matched healthy controls	V3-V4 16S rRNA sequencing	Goedert et al.^39^
Obese women showed higher Bacillota, *Faecalibacterium prausnitzii*, and *Blautia spp*. ratios compared to normal-weight patients. Stage II/III BC patients had higher Bacteroidota, *Clostridium coccoides*/*leptum* clusters, *Faecalibacterium prausnitzii*, and *Blautia spp*. than stage 0/I.	Fecal samples from 31 early-stage BC women (15 stage 0, 7 stage I, 7 stage II, 2 stage III; 90% ER/PgR+, 15% HER2+; 8 overweight).	16S rRNA sequencing	Luu et al.^83^
Postmenopausal BC patients had higher *Escherichia coli*, *Citrobacter koseri*, *Acinetobacter radioresistens*, *Enterococcus gallinarum*, and *Fusobacterium nucleatum*, but lower *Eubacterium eligens* and *Roseburia inulinivorans*.	Fecal samples from 18 premenopausal BC, 25 premenopausal healthy controls, 44 postmenopausal BC, and 46 postmenopausal healthy controls.	16S rRNA sequencing	Zhu et al.^162^
BC patients showed increased α-diversity and altered composition in IgA-positive and IgA-negative fecal microbiome.	Fecal and urine samples from 48 postmenopausal BC women (11 stage 0, 25 stage I, 10 stage II, 2 stage III; 88% ER+) and 48 matched healthy controls.	V4 16S rRNA sequencing	Goedert et al.^38^
Patients with prior chemotherapy had higher Actinomycetota and Bacteroidota linked to polyunsaturated fatty acids (PUFAs), while those without chemotherapy had lower *Bifidobacterium* linked to PUFAs.	Fecal and capillary blood samples from 124 individuals (123 women, 1 man; 46% with chemotherapy history).	V3-V4 16S rRNA sequencing; Gas chromatography for fatty acid composition	Horigome et al.^47^
BC patients with higher body fat had lower *Akkermansia muciniphila* (AM). Those with higher AM showed increased *Prevotella* and *Lactobacillus* and decreased *Campylobacter* and *Helicobacter* ratios.	Fecal samples from 32 BC women (stages 0 to II).	V4 16S rRNA sequencing	Frugé et al.^34^
At T_0_, the intervention group had lower *Clostridium* and higher *Escherichia*. At T_2_, probiotic-treated participants showed increased *Eubacterium* and *L-Ruminococcus*, reduced *Bacteroides* and *Butyricicoccus*, and a decreased Bacteroidota-to-Bacillota ratio.	34 BC survivors on a Mediterranean diet (16 with probiotics for 2 months, 18 without); microbiome analyzed at T_0_ and T_2_.	V3-V4 16S rRNA sequencing	Pellegrini et al.^105^
*Faecalibacterium* and *Bifidobacterium* were higher in premenopausal controls than postmenopausal controls. *Bifidobacterium* and *Akkermansia* were enriched in controls, while *Sutterella* and *Haemophilus* were higher in BC patients.	Fecal samples from 267 individuals (50 premenopausal controls, 17 postmenopausal controls, 100 premenopausal BC, 100 postmenopausal BC).	V3-V4 16S rDNA sequencing	Hou et al.^163^
The BN group had higher abundances of Pseudomonadota, *Staphylococcus*, *Campylobacter*, and *Moraxellaceae*, and lower *Paraprevotella* compared to NC. The BM group had dominant Pasteurellaceae, *Haemophilus*, *Planococcaceae*, *Lysinibacillus*, and Neisseria, while the BN group had *Megamonas*, *Lactobacillales*, *Bacilli*, *Streptococcus*, *Akkermansia*, and *Oxalobacter*. BM group showed higher Lactobacillales, Bacilli, *Veillonella*, *Streptococcus*, and *Campylobacter*, while lower *Megamonas*, *Clostridia*, *Akkermansia*, and *Paraprevotella* compared to NC.	Fecal samples from 25 NC, 32 BC without metastasis (BN), and 22 BC with bone metastasis (BM).	V4 16S rRNA sequencing	Wenhuiet al.^151^
BC patients had high *Prevotella*, *Porphyromonas*, *Peptoniphilus*, and *Megamonas*. BBLs had *Lactobacillus*, *Escherichia*, and *Coprobacillus*, while healthy controls had *Cloacibacillus*, *Asaccharobacter*, *Christensenella*, *Alistipes*, *Tissierella*, *Hydrogenoanaerobacterium*, *Butyricimonas*, *Acidaminococcus*, *Oxalobacter*, *Collinsella*, and *Eubacterium*.	Fecal samples from 26 BC patients, 20 with BBLs, and 20 healthy controls.	V3-V4 16S rRNA sequencing	Ma et al.^85^
Significant differences in phyla between BC survivors and healthy controls included Armatimonadota, Bacteroidota, Chloroflexota, Fibrobacterota, Bacillota, Gemmatimonadota, Nitrospirota, Marinimicrobia, Spirochaetota, Saccharimonadota, Verrucomicrobiota, and Latescibacterota. Bacteroidota and Bacillota were more abundant in group 2.	Samples from 23 BC survivors (group 1) and 291 healthy female controls (group 2).	V3-V4 16S rRNA sequencing	Caleça et al.^16^
Nine causal relationships were identified between the gut microbiome and total BC, with ten and nine causal relationships for ER- and ER+ BC, respectively. *Ruminococcaceae* and *Parabacteroides* were prominent across all categories, with *Desulfovibrio* expressed in ER- and total BC, and *Sellimonas*, *Adlercreutzia*, and *Rikenellaceae* in ER+ BC and total BC.	Data from the Breast Cancer Association Consortium (BCAC) involving 228,951 samples (122,977 BC cases and 105,974 controls).	Genome-wide association study (GWAS)	Hong et al.^46^
No significant differences in α-/β-diversity at the phylum and species level between responders (R) and non-responders (NR). ML algorithms identified *Bifidobacterium longum* and *Ruminococcus callidus* as discriminants for R, and *Clostridium innocuum* and *Schaalia odontolytica* for NR.	Fecal samples from 14 MBC patients classified as R and NR based on progression-free survival.	V3–V4 16S rRNA sequencing	Schettini et al.^124^

Several studies have demonstrated significant alterations in gut microbial diversity among breast cancer patients compared to healthy individuals. Goedert et al. observed a reduction in overall microbial diversity in breast cancer patients, characterized by an increase in *Clostridiaceae*, *Faecalibacterium*, and *Ruminococcaceae*, alongside a decrease in *Dorea* and *Lachnospiraceae*.^38,39^ Similarly, Ma et al. reported that *Prevotella*, *Porphyromonas*, *Peptoniphilus*, and *Megamonas* were enriched in breast cancer patients, whereas benign breast lesions were dominated by *Lactobacillus*, *Escherichia*, and *Coprobacillus*.^85^ In contrast, healthy individuals exhibited greater microbial diversity, with taxa such as *Cloacibacillus*, *Asaccharobacter*, *Christensenella*, *Alistipes*, *Tissierella*, *Hydrogenoanaerobacterium*, *Butyricimonas*, *Acidaminococcus*, *Oxalobacter*, *Collinsella*, and *Eubacterium* being prominent.^85^ Consistent with these findings, Caleça et al. observed that Bacteroidota and Bacillota were significantly more abundant in healthy controls, suggesting that specific microbial populations may either contribute to or protect against breast cancer development.^16^

Beyond compositional differences, variations in the gut microbiome have been observed across different stages of breast cancer. Bard et al. found that *Blautia* spp. were more abundant in early-stage breast cancer (Stage I) compared to later-stage disease (Stage III), while the relative abundance of *Bifidobacterium blautia* and *Faecalibacterium prausnitzii* varied significantly across clinical stages.^11^ Similarly, Luu et al. reported higher abundances of Bacteroidota, *Clostridium coccoides* cluster, *Clostridium leptum* cluster, *Faecalibacterium prausnitzii*, and *Blautia* spp. in stage II/III breast cancer patients compared to those in stage 0/1.^83^ Miko et al. further observed that microbial diversity is most reduced in the early phases of breast cancer, suggesting that shifts in the gut microbiome occur at the initial stages of tumorigenesis rather than as a secondary consequence of disease progression.^94^ These findings highlight the dynamic interaction between gut microbial composition and tumor development, reinforcing the possibility of microbiome-based biomarkers for early detection.

Distinct gut microbial profiles have also been linked to different breast cancer subtypes. Hong et al. identified nine causal relationships between specific gut microbes and breast cancer, with variations observed between estrogen receptor-positive (ER+) and estrogen receptor-negative (ER–) subtypes.^46^ A correlative study by Schettini et al. showed that certain bacterial species could predict responses to CDK4/6-inhibitors in metastatic breast cancer (MBC) patients.^124^ They observed no significant differences between responders and non-responders in terms of α-/β-diversity at the phylum and species level. However, machine-learning algorithms evidenced four bacterial species as a discriminant for responders (*Bifidobacterium longum*, *Ruminococcus callidus*) and non-responders (*Clostridium innocuum*, *Schaalia odontolytica*).^124^ These findings suggest that gut microbiome composition may influence treatment response, with potential implications for microbiome-based predictive models in personalized oncology.

Hormonal fluctuations associated with menopause may further shape gut microbial dynamics in breast cancer patients. Zhu et al. reported that postmenopausal breast cancer patients exhibited higher microbial diversity than premenopausal patients, with distinct taxonomic enrichments.^162^ Specifically, *Salmonella enterica, Actinomyces spp. HPA0247, Fusobacterium nucleatum, Shewanella putrefaciens, Enterococcus gallinarum, Citrobacter koseri, Shewanella putrefaciens*, and *Escherichia coli* were found to be more prevalent in the fecal microbiome of postmenopausal BC women. In contrast, there was no discernible difference between premenopausal patients and controls.^162^ These findings suggest that gut microbiome alterations may be more pronounced in postmenopausal breast cancer, potentially influenced by age-related metabolic and immunological changes.

Lifestyle factors, including diet and obesity, have been implicated in shaping the gut microbiome in breast cancer patients. Frugé et al. found that patients with higher body fat had lower levels of *Akkermansia muciniphila*,^34^ a bacterium linked to metabolic health. In a dietary intervention study, Pellegrini et al. examined the effects of a Mediterranean diet with or without probiotics in breast cancer survivors. They found that probiotic supplementation led to increased levels of beneficial microbes such as *Eubacterium*, *Lachnospiraceae*, and *Ruminococcus*, while reducing the abundance of Bacteroidota and *Butyricicoccus*.^105^ These findings suggest that dietary modifications and probiotic supplementation may serve as potential strategies to restore gut microbial balance and improve patient outcomes.

Experimental studies have further demonstrated the gut microbiome’s role in influencing treatment response across various breast cancer subtypes and clinical outcomes. Horgoime et al. observed that breast cancer patients with a history of chemotherapy had higher levels of Actinomycetota and Bacteroidota than those without chemotherapy.^47^ Wenhui et al. identified distinct taxa associated with metastatic and non-metastatic breast cancer groups, such as *Pasteurellaceae*, *Haemophilus*, *Planococcaceae*, *Lysinibacillus*, and *Neisseria* in metastatic cases and *Megamonas*, *Lactobacillales*, *Bacilli*, *Streptococcus*, *Akkermansia*, and *Oxalobacter* in non-metastatic cases.^151^ These findings highlight the potential role of gut microbiome profiling in predicting disease progression and guiding therapeutic decisions.

The accumulating evidence suggests that gut microbiome dysbiosis is intricately linked to breast cancer pathogenesis, influencing both estrogen-dependent and non-estrogen-dependent pathways.^116^ Through the regulation of circulating steroid hormones, immune function, and metabolic processes, the gut microbiome may act as either a risk factor or a protective element in breast cancer development.^147^ Notably, studies have reported a reduction in gut bacteria responsible for short-chain fatty acid (SCFA) production in breast cancer patients, indicating a possible role for these microbial metabolites in disease progression and immune modulation.^131^ However, despite these promising insights, variability in study populations, age, ethnicity, geographic location, sequencing methods, and analytical techniques limits the ability to draw definitive conclusions. A more comprehensive characterization of the breast cancer “oncobiome” is essential for advancing microbiome-targeted therapeutic strategies and improving patient prognosis.

## Gut microbiome-derived metabolites

The human gut microbiome is a complex ecosystem of microorganisms that plays a critical role in maintaining host health through diverse physiological processes. One of the primary mechanisms by which the gut microbiome interacts with the host is through the production of metabolites; small molecules that serve as intermediate or end-products of microbial metabolism.^2,68^ These metabolites are integral to numerous host functions, including immune system maturation, regulation of circadian rhythms, and maintenance of mucosal integrity.^77^ However, dysregulation of these metabolites has been implicated in a wide range of pathological conditions, such as neurological disorders,^139^ metabolic syndromes,^68^ and inflammatory bowel disease (IBD).^68^

Dietary factors are among the most significant modulators of gut microbiome composition and metabolite production. Emerging evidence suggests that dietary changes can profoundly influence the gut microbial ecosystem, thereby impacting the development or prevention of various disorders.^51^ For example, the fermentation of dietary fiber and resistant starch by gut microbes generates SCFAs, including butyrate, propionate, and acetate.^51^ These SCFAs are known for their anti-inflammatory properties and have been shown to play a protective role in diseases such as cancer.^27^ This underscores the potential of dietary interventions in modulating gut dysbiosis and promoting gut homeostasis.^27^

Beyond their role in maintaining physiological balance, gut microbial metabolites are increasingly recognized as key mediators linking the microbiome to host health and disease, particularly in the context of cancer progression.^155^ Their ability to influence host cellular processes and signaling pathways positions them as critical molecular bridges between microbial activity and systemic health. Consequently, gut microbial metabolites hold significant promise as early diagnostic biomarkers and therapeutic targets for metabolic diseases and beyond.^2^

The study of gut microbiome-derived metabolites not only provides insights into the etiology of various diseases but also opens new avenues for the development of innovative treatment strategies.^2,68,84,136,139^ By elucidating the complex interplay between microbial metabolites and host physiology, researchers can better understand disease mechanisms and design targeted interventions to restore microbial and metabolic balance.

## Gut metabolites in breast cancer progression and treatment

Gut microbiome-derived metabolites play a significant role in breast cancer development and progression through diverse mechanisms, including direct carcinogenic activity and modulation of cellular pathways involved in proliferation, apoptosis, and metastasis.^13^ One key mechanism by which the gut and breast microbiomes contribute to carcinogenesis is by altering hormonal signaling pathways. For instance, the gut microbiome can metabolize and secrete hormone-like bioactive substances, such as endogenous progesterone metabolites, prolactin, active androgens, and reactivated estrogens, which increase breast tissue exposure to hormonal triggers and influence breast cancer risk.^5^

The gut microbiome also contributes to breast cancer progression through the production of metabolites that induce oxidative stress or exert pro-carcinogenic effects. For example, lithocholic acid, a secondary bile acid, has been implicated in increased oxidative stress in breast cancer patients.^78^ Additionally, certain gut-derived metabolites, such as colibactin from *pks+ Escherichia coli* and Bacteroides fragilis toxin (BFT) from *Bacteroides fragilis*, produce pro-carcinogenic toxins that can damage breast tissue and enter systemic circulation. Conversely, other metabolites, including cadaverine,^63^ indoxylsulfate,^123^ and lithocholic acid^62^ have been shown to impede breast cancer progression, highlighting the complex and dual role of microbial metabolites in this disease.

While no specific therapies currently target gut metabolites for breast cancer treatment,^50^ this area holds significant therapeutic potential. Emerging evidence suggests that secondary bile acids, which are exclusively produced by gut bacteria, can influence breast cancer progression. For instance, studies in mice have demonstrated that secondary bile acids can induce a mesenchymal-to-epithelial transition, thereby reducing tumor aggressiveness, proliferation, and metastatic potential.^94^ These findings underscore the need for further research to identify specific metabolites that could serve as therapeutic targets.^50^

The pro-cancer and anti-cancer properties of gut-derived metabolites have been extensively explored over the past three decades. Table 2 summarizes key findings from studies selected based on keyword searches using terms such as “gut microbiome,” “gut microbiota,” “breast cancer,” “gut microbial metabolite,” “secondary metabolites,” “pro-cancer,” and “anti-cancer.” The assessment and exclusion criteria were as described above for studies in Table 1. Among these metabolites, nisin, a bacteriocin produced by *Lactococcus lactis*, has demonstrated antitumor efficacy against breast cancer.^8,50,103^ Similarly, inosine has been shown to induce apoptosis and inhibit breast cancer cell growth.^50,133^ Non-digestible carbohydrates, which modulate gut microbial composition, have also been found to influence the development of estrogen-dependent breast cancer tumors.^50^

**Table 2.** Studies assessing the possible anti-cancer and pro-cancer effects of key metabolites concerning breast cancer.

Metabolite Group	Metabolite	Potential Role in Breast Cancer	Effect	Reference
Bacteriocins	Nisin	Anticancer potential against MCF-7 breast adenocarcinoma cell line.	Anti-cancer	Avand et al.^9^
		Encapsulation in cyclodextrin-based nanosponges enhances stability and selective cytotoxicity against breast cancer cells (MCF-7).		Monfared et al.^97^
		Activation of mitochondrial apoptosis pathway, increased ROS production, and alterations in mitochondrial membrane potential leading to cell death.		Sadri et al.^118^
		Synergistic activity with doxorubicin at sub-inhibitory concentrations.		Monfared et al.^97^
Natural purine nucleoside	Inosine	Promotes cell proliferation and enhances tumor mitochondrial respiration.	Pro-cancer	Samami et al.^121^
		Stimulates cell division via adenosine receptors and may aid in tumor cell proliferation via purinergic receptors.		Zhang et al.^159^
		Demonstrated primary cytoprotective activities during breast cancer hypoxia, rather than adenosine, which was previously thought to be the primary compound responsible for this bioactivity.	Anti-cancer	Smith et al.^133^
Short-chain fatty acids	Butyrate	Inhibited tumorigenesis and tumor growth at higher concentrations, but at lower concentrations, it promotes carcinogenesis.	Pro-cancer	Chopin et al.^164^
		Inhibited cell proliferation in a dose-dependent manner with the IC50 value of 1.26 mM. Induced morphological changes to the MCF7 cells, and cell cycle arrest in the G1 phase.	Anti-cancer	Semaan et al.^127^
		Inhibited MCF7 cell viability in a dose- and time-dependent manner, decreased B-cell lymphoma 2 (Bcl-2) protein expression and induced morphological changes.		Wang et al.^149^
		Induced cytotoxicity and apoptosis in both breast cancer cell lines, and increased expression of 15-lipoxygenase type 1 (15-Lox-1) and production of 13-Hydroxyoctadecadienoic acid (13(S)HODE).		Salimi et al.^119^
		Initiated epigenetic changes to acetylation of proteins; pyruvate kinase activity was increased in MDA-MB-231 cells and lactate dehydrogenase activity was increased in T47-D cells. Increased oxygen consumption in the MDA-MB-231 and T47-D cell lines.		Rodrigues et al.^115^
		Inhibited cell proliferation in a dose- and time-dependent manner. Induced cell cycle arrest in the G1/G2 phase and a decrease in the S phase and caused chromatin relaxation.		Li et al.^72^
		Cell inhibition of 34% against MCF7 cells, increased histone H3K9 acetylation, and increased expression of p21waf1 and Retinoic acid receptor beta (*RARβ*).		Andrade et al.^6^
		Combined treatment of NaB and trastuzumab demonstrated synergistic growth inhibition and elevated mRNA and protein levels of p27Kip1.		Chen et al.^20^
		Induction of caspase-3, −10, and − 8, and formation of DNA fragmentation, in a dose- and time-dependent manner. Triggered apoptosis via the induction of caspase-10 activity.		Zhang et al.^165^
		Inhibited cell growth of MCF7 cells dose-dependently, induced cell cycle arrest in the G2/M phase, reduced p53 expression, decreased Bcl-2 mRNA and protein levels, increased apoptosis, and reduced glutathione levels.		Cui et al.^166^
		Induced cell cycle arrest and apoptosis via interaction with p21waf1/cip1 with cyclin-dependent kinase (CDK) and decreased proliferating cell nuclear antigen (*PCNA*) levels.		Chopin et al.^164^
		Increased the expression of tumor necrosis factor receptor 1 (TNF-R1) and receptor 2 (−R2), TRAIL receptor 1 (TRAIL-R1) and receptor 2 (−R2), and Fas in MCF7 cells and acted synergistically with these receptors to inhibit cell proliferation and induced apoptosis via p21waf1 and its interaction with *PCNA*.		Chopin et al.^167^
		Inhibited cell proliferation in all cell lines. Induced cell cycle arrest in the G2/M phase in MDA-MB-231 cells, and in the G1 phase for the other four cell lines. Inhibited cell growth in a p53-independent manner and induced apoptosis via the Fas/Fas L system.		Chopin et al.^168^
		Increased bioavailability when coupled with the hyaluronic acid drug delivery system due to the ability to bind to CD44 receptors, which are prominent on tumor surfaces.		Coradini et al.^169^
		Induced cell cycle arrest in the G2 phase via the inhibition of histone H1 kinase activities, and increased levels of p21waf1.		Lallemand et al.^170^
		Inhibitory effect of 85–90% with a dose- and time-dependent inhibition of cell proliferation, induced cell cycle arrest in the G2/M phase, resulting in the induction of apoptosis in the estrogen receptor-positive cell lines MCF7 and T47-D.		Coradini et al.^171^
		Initiated significant hyperacetylation of histones in MCF7 cells and lowered estrogen receptor levels.		Stevens et al.^172^
		Induced morphological changes in MCF7 cells and reduced cell proliferation.		Abe et al.^173^
	Acetate	Induces apoptosis in breast cancer cells by modulating pro-apoptotic (PARP, p53, Bax) and anti-apoptotic (Bcl-2, Bcl-xL) proteins.	Anti-cancer	Liew et al.^74^
		Causes cell cycle arrest at the G1/S checkpoint, inhibiting proliferation and limiting tumor growth.		Kwon et al.^65^
		Inhibits breast cancer cell migration and metastasis by downregulating focal adhesion kinase (FAK) and Akt signaling pathways.		Kwon et al.^65^
		Ethyl acetate extracts from natural sources (Orostachys japonicus, Chromolaena odorata) exhibit cytotoxic effects against breast cancer cells with effective inhibition of cell viability.		Yusuf et al.^156^
	Propionate	Inhibits the proliferation of MCF-7 and JIMT-1 breast cancer cell lines in a dose-dependent manner (5 to 20 mM).	Anti-cancer	Park et al.^104^
		Suppresses the JAK2/STAT3 signaling pathway, leading to cell cycle arrest at the G0/G1 phase and an increase in ROS, promoting apoptosis.		Park et al.^104^
		Enhances autophagic activity as a compensatory response to pro-apoptotic effects, though not fully counteracting antiproliferative actions.		Park et al.^104^
		Alters cyclin expression and induces cell cycle arrest in various breast cancer subtypes, aiding in therapeutic strategies.		Ibrahim et al.^49^
	Lactate	Modulates expression of oncogenes and cell cycle regulators in MCF7, leading to increased cell proliferation and survival.	Pro-cancer	San-Millán et al.^122^
		Associated with higher tumor grades, suggesting a link between lactate concentration and aggressive breast cancer phenotypes.		Cheung et al.^21^
		Activates GPR81, impairing immune responses by inhibiting cytotoxic T cells and promoting an immunosuppressive tumor microenvironment.		Li et al.^73^
		Contributes to extracellular acidosis, enhancing tumor cell survival and resistance to chemotherapy.		Xing et al.^153^
Bile acids metabolites	Lithocholic acid	Induces cancer cell death at supraphysiological concentrations. Modulates epithelial-to-mesenchymal transition (EMT), cancer cell metabolism, and proliferation.	Anti-cancer	Kovács et al.^62^ Mikó et al.^94^
		Increases oxidative and nitrosative stress by downregulating NRF2 and upregulating pro-oxidative enzymes, slowing down breast cancer cell proliferation.		Zhang et al.^158 174^
		Enhances pro-oxidant gene expression and oxidative stress markers, correlated with less aggressive forms of breast cancer.		Kovács et al.^62^
		LCA’s tumor-suppressive effects have been seen in breast cancer cell lines like MCF7.		Goldberg et al.^40^ Goldberg et al.^41^ Mikó et al.^94^
		Loss of the LCA-TGR5-oxidative stress pathway in advanced or triple-negative breast cancer (TNBC) cases is associated with worse clinical outcomes.	Pro-cancer	Kovács et al.^62^
Fatty Acids	Conjugated Linoleic Acids	CLAs reduce cell proliferation in breast cancer cell lines (MCF-7, MDA-MB-231).	Anti-cancer	Maggiora et al.^88^
		CLAs induce caspase-dependent apoptosis in colon tumor cells (SW 480) by altering caspase 3 and 9 activity, but their effect on breast cancer apoptosis needs further exploration.	Anti-cancer	Lampen et al.^66^
		CLAs influence the activity of estrogen receptors, potentially contributing to their antitumor effects in breast cancer cells.	Anti-cancer	Tanmahasamut et al.^140^ Wang et al.^148^
		CLAs suppress cell growth in breast cancer cell lines by altering PPARβ/δ and PPARα activities.	Anti-cancer	Maggiora et al.^88^
Polyphenols	Equol (from soy isoflavone daidzein)	Equol demonstrates anticancer activity through pathways such as Akt/FOXO3a and MEK/ERK, and inhibits tumor cell migration and invasion in breast cancer.	Anti-cancer	Lu et al.^79^
	Phenolic acids and urolithins (from anthocyanins and ellagitannins)	These metabolites show anti-proliferative effects in triple-negative breast cancer models.	Anti-cancer	Teixeira et al.^141^
	Genistein (soy isoflavone)	Genistein can reverse tamoxifen’s therapeutic effects in postmenopausal breast cancer models, suggesting a potentially inhibitory role at low concentrations.	Pro-cancer	Jones et al.^54^
Polyamines	Putrescine, Cadaverine, Spermidine, Spermine	Elevated polyamine concentrations in tumor tissues correlate with high metabolic activity in breast cancer, potentially promoting tumor growth.	Pro-cancer	Giardiello et al.^175^
	Polyamines (via RAD51 recombinase)	Polyamines enhance double-strand DNA repair by RAD51 recombinase, maintaining genomic integrity in breast cancer cells.	Anti-cancer	Lee et al.^69^
	Polyamines (via LSD1 inhibition)	Polyamines inhibit lysine-specific demethylase 1 (LSD1), implicated in tumor suppressor gene silencing, offering a potential therapeutic strategy in breast cancer.	Anti-cancer	Baylin et al.,^12^ Sharma et al.^129^
AhR ligands	Tryptamine, Skatole, Indole derivatives (IAcA, IA, IAld, ILA)	These microbial metabolites act as ligands for the aryl hydrocarbon receptor (AhR), which influences immune responses and tumor progression. Dysregulated AhR activity is linked to breast cancer progression.	Pro-cancer	Hubbard et al.,^48^ Zelante et al.^157 176^
	Urolithin A (derived from polyphenol breakdown)	Urolithin A acts as an AhR ligand with anti-inflammatory properties, reducing cytokines and prostaglandin expression, potentially inhibiting inflammation and cancer progression in breast cancer.	Anti-cancer	Muku et al.^98^
	Kynurenine, Kynurenic acid	These host-derived metabolites can influence immune evasion by tumors, suggesting a role in promoting immune tolerance and potentially contributing to breast cancer progression.	Pro-cancer	Hao et al.,^177^ Ji et al.^178^

One metabolite of particular interest is sodium butyrate, SCFA with dual roles as a histone deacetylase (HDAC) inhibitor and a regulator of cancer cell death. Depending on the dosage and duration of exposure, sodium butyrate can either promote or inhibit breast cancer growth.^50^ It has demonstrated promising potential in inducing ultrastructural changes, apoptosis^120,149^ and *in vitro* anti-tumor activity.^70^ When combined with other anti-cancer agents, sodium butyrate has shown enhanced therapeutic effects, particularly against triple-negative breast cancer.^6,20,72,101,115,119,127,149^

The complex interplay between gut-derived metabolites and breast cancer progression underscores the need for further research to elucidate their mechanisms of action and therapeutic potential. The following sections explore the pro-cancer and anti-cancer properties of these metabolites in greater detail.

## Nisin as a breast cancer preventive agent

Nisin, a polycyclic peptide composed of 34 amino acids, is the most widely produced bacteriocin by the gut microbiome and has demonstrated significant anti-cancer potential in various studies. Although its application in breast cancer remains underexplored, recent preclinical research highlights its promising therapeutic properties.^9,10,97,118^ Produced through fermentation by the Gram-positive bacterium *Lactococcus lactis*, nisin has garnered attention for its selective cytotoxicity against cancer cells, including the MCF-7 breast adenocarcinoma cell line.^9,97^ Notably, when combined with the conventional chemotherapeutic agent doxorubicin, nisin exhibited synergistic activity at sub-inhibitory concentrations, enhancing cytotoxic effects and suggesting its potential as an adjunct therapy.^9^ This synergy is further supported by in vitro studies demonstrating improved treatment outcomes in skin cancer patients following the co-administration of nisin and doxorubicin.^10^

The anti-cancer effects of nisin are primarily mediated through the activation of the mitochondrial intrinsic apoptosis pathway. This process involves increased production of reactive oxygen species (ROS) and alterations in mitochondrial membrane potential, ultimately leading to cancer cell death.^118^ Recent advancements in drug delivery systems have further enhanced nisin’s therapeutic potential. For instance, encapsulation of nisin in cyclodextrin-based nanosponges (CD-NSs) has been shown to improve its stability and selective toxicity toward cancer cells, including HT-29 colon and MCF-7 breast cancer cells.^97^ Compared to free nisin, CD-NS-encapsulated nisin exhibits more pronounced cytotoxic effects on malignant cells while maintaining significantly lower toxicity toward non-cancerous cells.^43^ This selective toxicity is attributed to nisin’s ability to form pores in the plasma membrane of cancer cells, facilitating calcium influx and triggering apoptosis.^9^

The unique pharmacological properties of nisin, coupled with its role in promoting host health, position it as a promising candidate for anti-cancer research.^118^ However, further studies are needed to fully elucidate its therapeutic potential. Future research should prioritize in vitro investigations across a broader range of breast cancer cell lines, particularly those with limited treatment options, such as triple-negative breast cancer. Additionally, animal models and preclinical studies should focus on understanding nisin’s synergistic effects with standard anti-cancer therapies and establishing its dose-response relationship. These efforts will provide critical insights for advancing nisin to in vivo and clinical studies, paving the way for its potential application in breast cancer prevention and treatment.^97^

## Inosine as a potential candidate in breast cancer therapy

Inosine, a naturally occurring purine nucleoside, has emerged as a molecule of interest in breast cancer due to its dual role in promoting tumor progression and enhancing anti-tumor immunity. Inosine contributes to breast cancer progression by stimulating cell proliferation and improving mitochondrial respiration in tumor cells.^121^ Research has shown that inosine, generated by dying and dead cells, stimulates cell division through adenosine receptors and may also facilitate tumor cell proliferation via purinergic receptors.^159^ Adenosine (ADO), a byproduct of adenosine triphosphate (ATP) hydrolysis, accumulates in the tumor microenvironment and can be converted to inosine. Inosine then binds to adenosine receptors (ARs), altering cell functions and activating intracellular signaling pathways through both receptor-dependent and receptor-independent mechanisms.^135^

The gut microbiome also plays a role in inosine production. *Bifidobacterium pseudolongum*, a commensal bacterium, produces inosine as part of its metabolic activities. The highest concentrations of inosine are found in the duodenum of the small intestine, with levels diminishing along the gastrointestinal tract.^87^ In vivo studies have demonstrated that systemic inosine concentrations in mice mono-colonized with *Bifidobacterium pseudolongum* primarily originate from inosine production in the upper gastrointestinal tract,^87^ highlighting the gut microbiome as a potential source of this metabolite.

Despite its role in tumor progression, inosine has shown promise in enhancing anti-tumor immunity, particularly in the context of immunotherapy. Preclinical studies suggest that inosine can increase tumor immunogenicity and improve the efficacy of immune checkpoint blockade (ICB) therapy.^159^ In vitro studies have demonstrated that inosine enhances T-cell-mediated tumor-killing activity, while in vivo studies have shown that it augments the effectiveness of checkpoint-blockade therapy.^121^ These findings position inosine as a potential adjuvant to enhance the response to immunotherapies.

Currently, clinical trials explicitly investigating inosine for breast cancer therapy are limited, and no active studies are directly focused on its application in this context. However, the preclinical evidence supporting its immunomodulatory effects warrants further exploration. If validated in clinical trials, inosine could be repurposed as a therapeutic agent to enhance patient responses to immunotherapies, particularly when used in combination with immune checkpoint inhibitors.^159^ Future research should focus on elucidating the mechanisms underlying inosine’s dual roles in tumor progression and anti-tumor immunity, as well as evaluating its safety and efficacy in clinical settings.

## Short-chain fatty acids

Short-chain fatty acids (SCFAs), including acetate, propionate, and butyrate, and lactate, are among the most abundant gut metabolites, primarily produced by bacterial species such as *Eubacterium rectale*, *Clostridium leptum*, and *Faecalibacterium prausnitzii*, and lactate-utilizing species like *Eubacterium hallii* and *Anaerostipes*.^52^ These metabolites play critical roles in modulating cancer hallmarks such as cell proliferation, apoptosis, and gene expression, making them key players in breast cancer progression.^93^ Below, we discuss the individual roles of SCFAs in breast cancer, focusing on their mechanism of action and therapeutic potential.

### Butyrate paradox in breast cancer

Butyrate, one of the most studied SCFAs, exhibits a dual role in cancer, often referred to as the “butyrate paradox.” At high concentrations, butyrate demonstrates potent anti-cancer effects, while at low concentrations, it may promote carcinogenesis.^33^ In the gastrointestinal tract, butyrate is produced by *Bacillota* through the fermentation of dietary fibers, with concentrations often exceeding 100 mM.^96^ Its anti-cancer properties are mediated through multiple mechanisms, including HDAC inhibition, modulation of apoptosis, cell cycle arrest, and epigenetic regulation.^96^ For instance, butyrate induces apoptosis in breast cancer cell lines (e.g., MCF7, T47-D, BT-20) via Fas-mediated pathways and upregulation of pro-apoptotic proteins like BAX.^18^ Furthermore, butyrate can arrest the cell cycle, especially in the G1 and G2/M phases, thereby inhibiting cell proliferation.^18^ It also improves radiation therapy’s efficacy when combined with anti-cancer agents.^43^ However, preclinical studies have highlighted its pro-tumoral effects at low concentrations.^33^ For instance, in an *in vitro* study, hepatocellular carcinoma (HCC) cells exhibited increased growth when exposed to low concentrations of NaB (<0.5 mM) compared to untreated controls (*p* < 0.05).^53^ Thus, emphasizing the need for careful dose optimization in therapeutic applications.^53^

Butyrate’s potential as a therapeutic agent is further supported by its synergistic effects with other treatments. For example, combining butyrate with trastuzumab (Herceptin) enhances anti-cancer activity in HER2-overexpressing breast cancer cells.^20^ The *in vitro* investigation found that co-administration of trastuzumab, together with increased levels of p27^Kip1^ mRNA and protein, increased the anti-cancer impact of NaB.^20^ Similarly, co-administration with retinoids or vitamin A has shown increased efficacy in inhibiting breast cancer cell proliferation.^6^ Despite its promise, butyrate’s poor bioavailability and pharmacokinetic limitations hinder its clinical use. Recent advancements in drug delivery systems, such as hyaluronic acid-based carriers and nano-delivery platforms, aim to overcome these challenges. Further clinical studies are needed to establish butyrate’s therapeutic potential and optimize its use in breast cancer treatment.^19,33^

### Acetate

Acetate, another key SCFA, exerts anti-cancer effects through apoptosis induction, cell cycle arrest, and inhibition of metastasis.^93^ In breast cancer cell lines (e.g., MDA-MB-231, MCF-7), acetate derivatives modulate pro-apoptotic (e.g., Bax, p53) and anti-apoptotic (e.g., Bcl-2, Bcl-xL) proteins, promoting cell death.^74^ Acetate also induces cell cycle arrest at the G1/S checkpoint, limiting tumor proliferation.^111^ Additionally, it inhibits metastasis by downregulating signaling pathways such as focal adhesion kinase (FAK) and Akt, which are critical for cell migration.^65,74^ Experimental studies have demonstrated the cytotoxic effects of acetate extracts from natural sources, such as *Orostachys japonicus* and *Chromolaena odorata*, against breast cancer cells, highlighting its therapeutic potential.^156^

### Propionate

Sodium propionate (SP) has shown significant anti-cancer activity in breast cancer, particularly through its effects on cell proliferation and apoptosis. SP inhibits the proliferation of breast cancer cell lines (e.g., MCF-7, JIMT-1) in a dose-dependent manner by suppressing the JAK2/STAT3 signaling pathway, inducing cell cycle arrest at the G0/G1 phase, and increasing reactive oxygen species (ROS) production.^104^ It also enhances autophagic activity as a compensatory response, though this does not fully counteract its anti-proliferative effects.^104^ These findings suggest that sodium propionate could serve as a promising candidate for breast cancer therapy by targeting critical signaling pathways involved in tumor growth and survival.^49,93,104,156^

### Lactate

Lactate, often regarded as a metabolic byproduct, has emerged as an oncometabolite with significant implications in breast cancer progression.^93^ It has been demonstrated to modulate the expression of key oncogenes and cell cycle regulators in breast cancer cell lines, such as MCF7, leading to increased cell proliferation and survival.^122^ Elevated lactate levels have been associated with higher tumor grades, suggesting a correlation between lactate concentration and aggressive breast cancer phenotypes.^21^ Lactate promotes an immunosuppressive tumor microenvironment by activating G protein-coupled receptors (GPR81), which inhibit cytotoxic T cells and enhance tumor cell survival.^73^ Additionally, lactate accumulation contributes to extracellular acidosis, further supporting tumor cell resistance to chemotherapy.^153^ These findings highlight the potential of targeting lactate metabolism as a therapeutic strategy to disrupt its tumor-promoting effects and restore immune responses in breast cancer.

## Lithocholic acid

Lithocholic acid (LCA), a secondary bile acid metabolite derived from the gut microbiota, has emerged as a promising anti-cancer agent with diverse effects on breast cancer progression.^40,41,62,82,94^ LCA exerts its anti-cancer effects by modulating several cancer hallmarks, including epithelial-to-mesenchymal transition (EMT), cancer cell metabolism, proliferation, and anti-cancer immunity.^62,94^ At supraphysiological concentrations, LCA induces cancer cell death, highlighting its therapeutic potential.^62,94^ Recent studies have further demonstrated that LCA increases oxidative and nitrosative stress at physiologically relevant tissue concentrations, mediated by the downregulation of NRF2, a key antioxidant transcription factor, and the upregulation of pro-oxidative enzymes.^158^ This imbalance in pro-oxidant and antioxidant systems generates reactive species that damage proteins and lipids, ultimately slowing breast cancer cell proliferation.^158^

While oxidative stress has been linked to modulating EMT and cellular metabolism in breast cancer, LCA-induced reactive species were insufficient to significantly impact these processes in certain models, suggesting the involvement of alternative pathways.^158^ For instance, LCA may influence the hypoxic response via hypoxia-inducing factors,^158^ mTORC1 signaling,^25^ or proteostasis pathways.^76^ Additionally, free radical production induced by LCA could potentially reprogram breast cancer cells, shifting them from cancer stem cells to tumor stroma cells, as suggested in related studies.^62,81,94^ The dual role of oxidative stress in breast cancer progression remains complex and context-dependent.^45^ Reactive species can act as both pro-carcinogenic agents, driving DNA damage and mutations,^22,36,37,112^ and anti-carcinogenic agents,^23,81,108^ inducing lipid peroxidation associated with improved survival.^62^ In this context, LCA’s ability to increase pro-oxidant gene expression and oxidative stress markers has been correlated with less aggressive, clinically benign forms of breast cancer.^62^ Conversely, the loss of the LCA – TGR5–oxidative stress pathway in advanced or triple-negative breast cancer (TNBC) cases is associated with worse clinical outcomes. Higher expression of pro-oxidant genes and CAR (constitutive androstane receptor) expression correlated with better survival, although this benefit was not observed in TNBC.^62^ These findings highlight the potential cytostatic and therapeutic role of LCA-mediated oxidative stress in breast cancer management.

Beyond breast cancer, LCA’s tumor-suppressive effects extend to other cancer types. Prior studies have shown that LCA induces cell death in neuroblastoma, prostate cancer, and MCF7 cells, underscoring its broad anti-cancer potential.^40,41,94^ Importantly, LCA fosters an anti-cancer milieu within the breast tumor microenvironment, selectively inhibiting breast cancer cell proliferation without affecting primary, non-cancerous cells.^94^ This specificity enhances its appeal as a therapeutic agent. Collectively, these studies position LCA as a key microbiota-derived metabolite with significant potential to modulate the tumor microenvironment and improve clinical outcomes in breast cancer.^23,25,36,40,41,45,62,76,81,82,94,108,112,158^

## Conjugated linoleic acids

Conjugated linoleic acids (CLAs), a group of positional and geometric isomers of linoleic acid, are recognized as functional food components due to their numerous health benefits, including their role in maintaining body composition and influencing energetic metabolism.^24^ Beyond their nutritional value, CLAs have garnered significant attention for their anticarcinogenic properties, with substantial evidence supporting their potential in cancer prevention and therapy.^71^

In vitro studies have demonstrated that CLAs reduce cell proliferation in a variety of cancer cell lines, including human colon cancer cells (HN-29, Caco-2),^66^ hepatoma cells (HepG2, SK-HEP-1), prostate cancer cells (LNCaP, PC3), bladder tumor cells (639 V, SG65), and breast cancer cells (MCF-7, MDA-MB-231).^88^ The antiproliferative effects of CLAs are associated with the modulation of peroxisome-proliferator-activated receptors (PPARs). Specifically, CLAs decrease PPARβ/δ activity while increasing PPARα activity, which contributes to their growth-suppressive effects.^66,88^ Additionally, CLAs influence the APC-β-catenin-TCF4 signaling pathway, leading to the suppression of genes involved in cell proliferation, such as c-myc, cyclin D1, and c-jun, in colonocytes.^66^ Beyond their antiproliferative properties, CLAs can induce caspase-dependent apoptosis in colon tumor cells (SW 480). This apoptotic effect is mediated by alterations in caspase 3 and 9 activity, increased membrane annexin V levels, and reduced expression of the anti-apoptotic protein Bcl-2.^66^ These mechanisms highlight the ability of CLAs to not only inhibit cancer cell growth but also promote programmed cell death.

In the context of breast cancer, CLAs have been shown to affect estrogen receptor activity, potentially contributing to their antitumor effects.^140,148^ However, whether CLAs directly interact with estrogen receptors or exert their effects through indirect mechanisms remains unclear. Further research is needed to elucidate the precise molecular pathways through which CLAs influence estrogen receptor signaling and their broader implications for breast cancer therapy. Collectively, these findings underscore the potential of CLAs as functional food components with significant anticarcinogenic properties. Their ability to modulate key signaling pathways, induce apoptosis, and influence estrogen receptor activity positions them as promising candidates for further investigation in cancer prevention and treatment.

## Polyphenols metabolites

Polyphenols are a diverse group of hydroxylated phenyl-containing compounds widely present as secondary metabolites in fruits, vegetables, seeds, herbs, and beverages.^110,146^ These dietary compounds have garnered significant attention due to their roles in modulating human health and their associations with cancer,^161^ diabetes,^17^ obesity,^92^ cardiovascular,^58^ and neurodegenerative diseases.^89^ Notably, higher plasma concentrations of polyphenol metabolites have been correlated with a reduced risk of breast cancer^106^ and colorectal adenomas.^64^

The interaction between polyphenols and gut microbiota is bidirectional. On one hand, polyphenols can modulate the composition and activity of the gut microbiota.^126^ On the other hand, gut bacteria metabolize polyphenols into bioactive compounds that exert systemic effects on the host.^29,57,80,142^ Most polyphenols escape digestion in the small intestine and reach the large intestine, where they are metabolized by gut bacteria.^102,144,152^ Key bacterial genera involved in polyphenol metabolism include *Clostridium sp*. and *Eubacterium sp*., along with probiotics like *Bifidobacterium sp*. and *Lactobacillus sp*..^126^ These bacteria convert polyphenols into a limited set of aromatic metabolites, such as phenolic acids and urolithins, which influence host physiology and contribute to their health benefits.^15,28^

Polyphenol metabolites exhibit anticarcinogenic properties through mechanisms such as cell cycle modulation, induction of apoptosis,^4,137^ inhibition of proinflammatory cytokines,^67^ and interactions with estrogen receptors.^86,128^ For example, equol, a bacterial metabolite derived from soy isoflavone daidzein, demonstrates anti-cancer activity through pathways such as Akt/FOXO3a^79^ and MEK/ERK.^99^ Equol also inhibits tumor cell migration and invasion via downregulation of matrix metalloproteinase-2 (MMP-2), matrix metalloproteinase-9 (MMP-9), and urokinase-type plasminogen activator (uPA).^160^ Similarly, phenolic acids and urolithins, metabolites of anthocyanins and ellagitannins, have shown anti-proliferative effects in triple-negative breast cancer models,^141^ further highlighting their therapeutic potential.

However, the effects of polyphenols and their metabolites on cancer are not uniformly beneficial. Some polyphenols can interfere with cancer therapies, complicating their use in clinical settings. For instance, genistein, a soy isoflavone, has been shown to reverse the therapeutic effects of tamoxifen at low concentrations in a postmenopausal breast cancer model.^54^ Additionally, certain polyphenols have been associated with mixed outcomes in cancer risk. While genistein has shown a slight protective effect against prostate cancer, other compounds like campesterol and stigmasterol have been positively associated with increased prostate cancer risk, though findings remain inconclusive.^138^

The dual effects of polyphenol metabolites in cancer, both beneficial and inhibitory, underscore the complexity of their role in cancer. Further research is needed to elucidate the mechanisms underlying their metabolism by gut microbiota and their interactions with host pathways. A deeper understanding of this interplay may pave the way for dietary interventions and therapeutic strategies in cancer management, offering new avenues for prevention and treatment.

## Polyamines

Polyamines (PAs), including putrescine, cadaverine, spermidine, and spermine, are microbial metabolites synthesized predominantly by Firmicutes in the gut.^44,90^ These compounds are also produced by host cells and can be absorbed from dietary sources in the upper intestine.^95^ As polycationic molecules, PAs bind to negatively charged macromolecules such as nucleic acids and membrane phospholipids, influencing a wide range of cellular processes.^125^ In bacteria, PAs play critical roles in maintaining cell wall stability,^31,61^ synthesizing siderophores,^42^ and protecting against free radicals^55^ and acidic environments.^114^ These microbial functions, combined with their host-cell interactions, position PAs as key metabolites in the gut microbiome-metabolite axis.

Dysregulated PA metabolism has been implicated in various cancer types, including breast cancer. Elevated intracellular PA levels and increased biosynthesis are commonly observed in highly proliferative cells, such as regenerating tissues and tumors.^117^ The enzyme ornithine decarboxylase (ODC), which catalyzes the rate-limiting step in PA biosynthesis, is often overactive in cancer cells. Elevated ODC activity correlates with increased PA concentrations in tumor tissues, a hallmark of colorectal and potentially breast cancers.^150^ Conversely, probiotic bacteria such as *Bifidobacterium* sp., *Lactobacillus* sp., and *Streptococcus* sp. can reduce PA levels in the gut by decreasing ODC activity and promoting apoptosis in colonic cells.^75^ These findings suggest that modulating PA metabolism through gut microbiome interventions, such as probiotic supplementation, may help reduce cancer risk.

In breast cancer, the high metabolic activity and elevated PA concentrations in tumor tissues present both a challenge and an opportunity for therapeutic interventions. PAs are essential for maintaining genomic integrity, as they enhance double-strand DNA repair by RAD51 recombinase^69^ and inhibit histone demethylases like lysine-specific demethylase 1 (LSD1), which are implicated in DNA hypermethylation and tumor suppressor gene silencing.^12,129^ These roles make PAs attractive targets for chemotherapeutic strategies aimed at disrupting cancer cell proliferation and survival.

Despite their potential anti-cancer roles, the impact of microbially synthesized PAs on breast cancer remains underexplored. While some studies suggest that PA metabolism by probiotic bacteria may contribute to anti-cancer effects, others highlight the proliferative potential of these metabolites in tumor cells. This duality underscores the complexity of PA biology in cancer and the need for further research to elucidate their precise mechanisms and therapeutic potential. Integrating insights into PA metabolism with gut microbiome studies may uncover novel therapeutic approaches for breast cancer. For instance, targeting tumor-associated polyamine dynamics through dietary interventions, probiotics, or pharmacological agents could enhance the efficacy of existing cancer treatments. Further research is crucial to fully understand the interplay between bacterial PA production and host cancer biology, paving the way for innovative strategies in breast cancer management.

## Aryl hydrocarbon receptor ligands

The gut microbiome plays a critical role in metabolizing dietary polyphenols and tryptophan (Trp) into bioactive compounds, including tryptamine, skatole, and indole derivatives (such as indole-3-acetic acid [IAcA], indole [IA], indole-3-aldehyde [IAld], and indole-3-lactic acid [ILA]). These metabolites act as ligands for the aryl hydrocarbon receptor (AhR), a transcription factor expressed in immune and epithelial cells.^48,59,157^ AhR ligands, along with host-derived metabolites like kynurenine and kynurenic acid, play a pivotal role in regulating immune responses, maintaining mucosal barrier function, and supporting metabolic homeostasis. However, dysregulated AhR activity has been implicated in inflammation-driven carcinogenesis, including processes such as epithelial-to-mesenchymal transition (EMT) and tumor cell dissemination, which are highly relevant to breast cancer progression.^3,91^

Emerging evidence suggests that cancer cells can exploit AhR signaling through Trp catabolism to produce immunosuppressive metabolites, enabling them to evade immune detection and promote tumor survival.^154^ For example, the kynurenine pathway, a major Trp catabolic route, generates metabolites that suppress anti-tumor immunity and foster a tumor-permissive microenvironment. Conversely, certain microbial metabolites, such as urolithin A (a product of polyphenol breakdown) act as AhR ligands with anti-inflammatory properties. Urolithin A has been shown to reduce pro-inflammatory cytokines like IL-6 and inhibit prostaglandin expression, highlighting its potential to counteract inflammation-driven carcinogenesis.^98^

The role of AhR ligands in cancer is complex and context-dependent, exhibiting both pro- and anti-cancer effects. In breast cancer, the high concentrations of AhR ligands in the tumor microenvironment suggest a significant yet underexplored influence on disease progression. For instance, while some AhR ligands may promote tumor growth and immune evasion, others, like urolithin A, may exert protective effects by modulating inflammation and immune responses. This duality underscores the need for further research to elucidate the precise mechanisms by which gut microbiome-derived AhR ligands influence breast cancer pathogenesis.

## Gut metabolites and standard chemotherapies

The gut microbiome and its metabolites play a pivotal role in modulating the effectiveness of standard chemotherapy, influencing both treatment outcomes and side effects.^155^ Gut microbial metabolites can remodel the tumor microenvironment, regulate key signaling pathways in cancer cells, and impact the efficacy and safety of chemotherapeutic agents.^155^ Emerging research has demonstrated a direct connection between the composition of the gut microbiome and the success of chemotherapy, highlighting the importance of a healthy gut microbial population in optimizing treatment outcomes.^13,132^ Conversely, chemotherapy administration can disrupt the gut microbiome, leading to microbial dysbiosis, which may negatively affect treatment efficacy and contribute to adverse clinical outcomes.^1^ These findings underscore the bidirectional relationship between the gut microbiome and chemotherapy, emphasizing the need to consider microbial health in cancer treatment strategies.

Recognizing the significance of the gut microbiome in chemotherapy outcomes, recent reviews have proposed modulating the gut microbiome as a complementary strategy to enhance the therapeutic potential of existing anti-cancer drugs.^145,155^ This approach involves manipulating the gut microbial community by boosting beneficial species, depleting harmful ones, or introducing missing microbial populations that may aid in cancer treatment.^145^ This manipulation may involve boosting or depleting specific microbial species and introducing missing communities that could aid in cancer treatment.^145^ For example, restoring gut microbiota balance and reducing systemic estrogen levels has been suggested as a therapeutic strategy to lower the risk and progression of breast cancer.^30^ Such interventions could improve the efficacy of chemotherapy while mitigating its side effects, offering a promising avenue for personalized cancer therapy.

The interplay between the gut microbiome and chemotherapy-derived metabolites has been extensively studied in the context of breast cancer. Research suggests that the gut microbiome can both enhance and diminish the efficacy of neoadjuvant therapy, depending on its composition and metabolic activity.^107^ For instance, gut microbiome-derived metabolites, such as SCFAs, have been shown to influence cancer treatment response and toxicity.^52^ SCFAs, produced through the fermentation of dietary fiber by gut bacteria, exhibit anti-inflammatory and immunomodulatory properties that may enhance the effectiveness of chemotherapy while reducing treatment-related toxicities.

Despite these promising findings, further research is needed to fully understand the potential benefits of combining gut microbial metabolites with standard anti-cancer drugs. Investigating the synergistic effects of these metabolites and chemotherapeutic agents could unlock new possibilities for cancer treatment, particularly in improving therapeutic outcomes and reducing adverse effects. Future studies should focus on identifying specific microbial metabolites and their mechanisms of action, as well as exploring strategies to modulate the gut microbiome in a targeted and clinically relevant manner.

## Challenges in targeting gut metabolites for breast cancer treatment

While gut metabolites hold significant promise as potential therapeutic targets for breast cancer, several challenges must be addressed to translate this potential into clinical applications. One major challenge lies in the complexity of the gut microbiome and its associated metabolites. The gut microbiome functions as a dynamic ecosystem, and the metabolites it produces can have diverse effects, ranging from beneficial to harmful, depending on the context^50^ Therefore, it is crucial to identify and target specific metabolites that are directly linked to breast cancer development and progression, while minimizing unintended effects on host health.

Another significant hurdle is the inherent diversity in gut microbiome composition and metabolite production among individuals, particularly those with different subtypes of breast cancer.^78^ This variability underscores the need for personalized approaches to target gut metabolites effectively. Precision medicine, which already considers genetic and molecular profiles in oncology, must now integrate microbiome data to optimize treatment strategies. This shift could usher in a new era of cancer therapy, where the microbiome is used as a tool for prognostication and personalized treatment planning.

Targeting gut metabolites also raises concerns about their potential influence on therapeutic efficacy and side effects. The gut microbiome can modulate the response to breast cancer treatments, including chemotherapy and immunotherapy, by altering drug metabolism, immune responses, and the tumor microenvironment.^5^ Therefore, when designing targeted therapies, it is essential to consider the potential interactions between gut metabolites and conventional treatments. For instance, certain metabolites may enhance treatment efficacy, while others could exacerbate side effects or promote drug resistance.

Addressing these challenges requires a multidisciplinary approach that combines microbiome research, metabolomics, and oncology. Future studies should focus on identifying specific gut metabolites associated with breast cancer, understanding their mechanisms of action, and developing strategies to modulate them in a targeted and personalized manner. By overcoming these challenges, researchers can unlock the full potential of gut metabolites as therapeutic tools, paving the way for more effective and personalized breast cancer treatments.

## Conclusion and future directions

Breast cancer is a multifaceted disease influenced by a combination of factors, including age, genetics, lifestyle, pregnancy history, and environmental exposures such as antibiotics. Surprisingly, nearly 70% of breast cancer cases lack identifiable risk factors beyond being female and over the age of 50. Recent research has shed light on the potential role of the microbiome, particularly the gut microbiome, in modulating local and systemic immune responses that may influence breast cancer development and progression. To fully understand the microbiome’s role in breast cancer, comprehensive research utilizing animal models, retrospective and prospective clinical studies, and well-designed clinical trials is essential. One promising avenue for future research is the interaction between the gut microbiome and the host immune system, as well as the microbial effects on host DNA. These interactions could pave the way for novel treatment strategies that target the microbiome to enhance immune responses or mitigate DNA damage. Additionally, the composition of the gut microbiome and its metabolites hold potential as biomarkers for breast cancer risk, progression, and treatment response. Investigating these biomarkers could enable earlier detection and more personalized therapeutic approaches.

Another critical area of exploration is the use of prebiotics, probiotics, and dietary supplements to restore a healthy gut microbiome balance. These interventions could serve as preventive measures or adjunct therapies for breast cancer, potentially improving treatment outcomes and reducing side effects. However, several challenges must be addressed, including the complexity of gut microbial communities, individual variations in microbiome composition and metabolite production, and potential interactions between gut metabolites and conventional breast cancer therapies. Overcoming these obstacles will require a multidisciplinary approach that integrates microbiome science, metabolomics, and oncology. Despite significant progress in linking gut microbial metabolites to health benefits, substantial gaps remain in understanding their therapeutic potential, either as standalone treatments or in combination with existing therapies. Future research should focus on elucidating the dose-response relationships between gut metabolites and breast cancer progression, as well as their mechanisms of action. Such insights are crucial for advancing gut metabolites into animal models and clinical trials, ultimately transforming cancer treatment paradigms.

Gut microbial metabolites represent a promising, cost-effective, and highly personalized alternative or complement to standard chemotherapy. By offering improved health benefits with fewer side effects, they could revolutionize breast cancer treatment. A deeper understanding of the gut microbiome’s role in breast cancer will not only enhance our ability to prevent and treat the disease but also open new avenues for precision medicine tailored to individual patients’ unique microbial and metabolic profiles.

## Data Availability

Data sharing is not applicable to this article as no new data were created or analyzed in this study.

## Highlights

- Gut microbial metabolites, such as Nisin, Sodium butyrate, Inosine, and others, are associated with breast cancer but our understanding of their association, especially with progression and breast cancer treatment, remains limited.
- Advances in next-generation sequencing (NGS) technologies, including metagenomics, have contributed significantly to deconvoluting this connection, however, more efforts are required to comprehensively characterize the anti-cancer potential of these gut metabolites.
- We have highlighted major microbial species and associated metabolites currently known to have a link with breast cancer and have been identified using metagenomic NGS.
- This resource may help the scientific community in further exploring the potential of gut metabolites as novel therapeutic targets for improved treatment and management of breast cancer patients by serving as a knowledge base.
